# Unraveling the Epigenetic Regulation of Regulatory T Cells in Cancer Immunity

**DOI:** 10.3390/cells15030228

**Published:** 2026-01-25

**Authors:** Kalpana Subedi, Nirmal Parajuli, Xzaviar Kaymar Solone, Jeffrey Cruz, Sahil Kapur, Deyu Fang, Qing-Sheng Mi, Li Zhou

**Affiliations:** 1 Center for Cutaneous Biology and Immunology Research, Department of Dermatology, Henry Ford Health, Detroit, MI 48202, USA; ksubedi1@hfhs.org (K.S.); nparaju1@hfhs.org (N.P.); xsolone1@hfhs.org (X.K.S.); jcruz12@hfhs.org (J.C.); skapur2@hfhs.org (S.K.); 2 Immunology Research Program, Henry Ford Cancer Institute, Henry Ford Health, Detroit, MI 48202, USA; 3 Department of Pathology, Northwestern University Feinberg School of Medicine, Chicago, IL 60611, USA; fangd@northwestern.edu; 4 Department of Medicine, College of Human Medicine, Michigan State University, East Lansing, MI 48824, USA; 5 Department of Medicine, Henry Ford Health, Detroit, MI 48202, USA; 6 Henry Ford Health + Michigan State University Health Sciences, Detroit, MI 48202, USA

**Keywords:** Tregs, epigenetics, histone modification, DNA methylation, microRNA, cancer

## Abstract

Regulatory T cells (Tregs) are central mediators of immune tolerance, yet within tumors they adopt specialized phenotypes that confer the potent suppression of anti-tumor immune responses. Emerging evidence indicates that this functional plasticity is not driven by genetic alterations but instead arises from dynamic and context-dependent epigenetic reprogramming. While individual epigenetic mechanisms controlling Treg development and stability have been described, how tumor-derived cues reshape Treg epigenetic states, how these programs differ across cancer types, and which features distinguish tumor-infiltrating Tregs from their peripheral counterparts remain incompletely understood. In this review, we synthesize recent advances in DNA methylation, histone modifications, chromatin accessibility, and non-coding RNA regulation that govern Treg identity and function with a particular emphasis on tumor-specific epigenetic adaptations. We highlight emerging epigenetic hallmarks of intratumoral Tregs, discuss unresolved mechanistic questions, and evaluate the therapeutic potential and limitations of targeting epigenetic pathways to selectively modulate Tregs in cancer. By integrating mechanistic, cancer-specific, and translational perspectives, this review aims to provide a conceptual framework for understanding how epigenetic regulation shapes Treg behavior in the tumor microenvironment and how it may be exploited for cancer immunotherapy.

## 1. Introduction

Regulatory T cells (Tregs) were first recognized as a distinct suppressive lineage in 1995, when Sakaguchi and colleagues demonstrated that removal of the CD4^+^CD25^+^ subset precipitated organ-specific autoimmunity, establishing their essential role in maintaining self-tolerance [1]. A major leap followed in 2001 with the identification of the scurfy gene, later named Foxp3, by Brunkow and Ramsdell, who showed that its disruption caused fatal lymphoproliferative autoimmunity in mice, defining Foxp3 as a master transcriptional regulator of this lineage [2]. In the same year, human genetic studies by Bennett, Brunkow, and Ramsdell demonstrated that Foxp3 mutations underlie immune dysregulation, polyendocrinopathy, enteropathy, and X-linked (IPEX) syndrome, confirming Foxp3 as the lineage-defining factor for Tregs in humans [3]. In honor of these foundational discoveries, which reshaped our understanding of immune self-restraint and opened new therapeutic avenues, Shimon Sakaguchi, Mary E. Brunkow and Fred Ramsdell were awarded the 2025 Nobel Prize in Physiology or Medicine.

Tregs are essential for maintaining immune tolerance and homeostasis, where they prevent autoimmunity and help regulate chronic inflammation [4]. While they are crucial for sustaining self-tolerance and overall immune balance, Tregs can also inadvertently suppress beneficial immune responses, including anti-tumor immunity [5]. Under normal physiological conditions, they control inflammation through coordinated mechanisms of peripheral tolerance and immunosuppression. They employ several strategies to suppress immune activity, including the production of inhibitory cytokines (e.g., IL-10, transforming growth factor-β: TGF-β, and IL-35), cytolysis via granzyme A/B and perforin, metabolic disruption (such as IL-2 consumption and the generation of adenosine and cyclic AMP), and the modulation of dendritic cell function through Ctla-4 and Lag3 interactions, which inhibit dendritic cell maturation [4,6]. These mechanisms primarily target effector T cells, dendritic cells, and other antigen-presenting cells, regulating immune responses to maintain balance.

**Figure 1:**
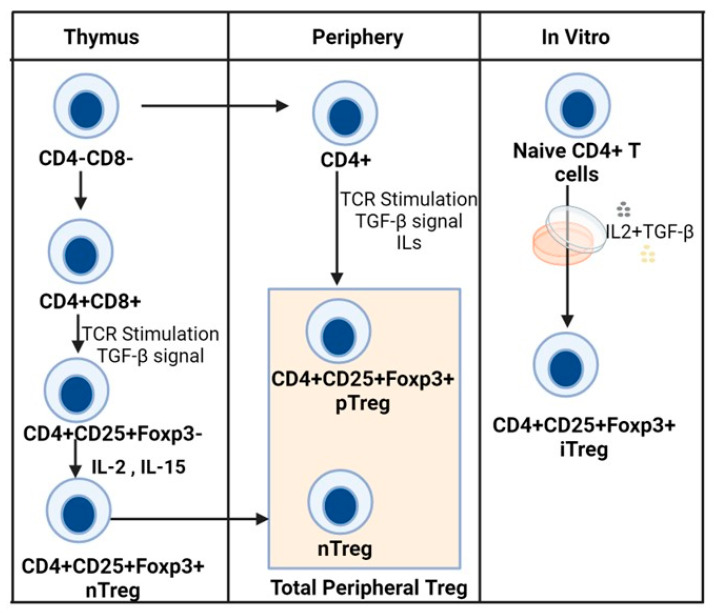
Schematic diagram of Tregs development. The peripheral Treg cell pool is composed of 2 distinct populations, natural Tregs (nTreg) and peripheral Tregs (pTregs). nTreg cells differentiate in the thymus, while pTreg cells are generated in lymphoid organs. In vitro-generated Tregs are called inducible Tregs (iTregs).

Tregs are a heterogeneous population of CD4^+^ T cells determined by their high expression of CD25 and low expression of CD127. The Foxp3 is a major transcription factor that is responsible for Tregs’ function and stability [7,8,9,10,11,12]. Foxp3 is a member of the forkhead transcription factor family and is primarily localized within the nucleus. Foxp3 plays a dual role as both a transcriptional activator and repressor of its target genes in Treg cells. In addition to Foxp3, there are other signature genes associated with Tregs, such as *Il2ra* (CD25), *Ctla4* (CD152), *Tnfrsf18* (GITR), *Ikzf2* (Helios), and *Ikzf4* (Eos), which play vital roles in Tregs’ function [13,14,15,16]. Foxp3-positive Treg cells can be categorized into different subpopulations based on their origin: thymus-derived Tregs (nTregs), peripheral-induced Tregs (pTregs), and in vitro-induced Tregs (iTregs) originating from CD4^+^CD25^−^ naive T cells (Figure 1). nTregs originate from the thymus and possess the necessary transcriptional and epigenetic features to persist as a self-renewing population in the periphery. Tregs’ development in the thymus entails the establishment of a specific epigenetic profile, which is distinct from, yet complementary to, Foxp3 expression, and it is necessary for specifying the Treg cell lineage identity and function. In contrast, induced Tregs differentiate in peripheral tissues from conventional CD4^+^ T cells, often in response to foreign antigens, allergens, or commensal microbes in the presence of TGF-β and IL-2. Both natural and induced Tregs are characterized by the presence of factors such as Helios and Neuropilin-1, which are thought to be specific to Tregs originating in the thymus [17,18,19]. Both pTreg and nTreg cells demonstrate high stability in the expression of Foxp3 and other Treg signature genes, allowing them to maintain their immune-suppressive functions. Treg cell identity and function become unstable when there is a loss of Foxp3 expression or alterations in the epigenetic landscape. Based on their functional state, Tregs can be classified into central Tregs (cTregs) characterized by CD44^low^CD62L^high^ and effector Tregs (eTregs) characterized by CD44^high^CD62L^low^. Tregs heavily infiltrate into tumors, which are called tumor-infiltrating Tregs (TI-Tregs). The immunosuppressive function of TI-Tregs supports the immune escape and tumor progression. While various factors contribute to Treg lineage stability, including surface protein markers and genomic epigenetic modifications, accumulating evidence supports the pivotal role of the Foxp3 transcription factor as a dominant regulator in Treg cell development and function maintenance.

Structurally, Foxp3 consists of three key domains: a proline-rich N-terminal domain (1–97 aa), a central zinc-finger leucine-zipper domain (98–260 aa) and a conserved C-terminal forkhead domain (337–423 aa) responsible for transcriptional activation and repression, oligomer formation and DNA binding, respectively [20]. These domains collaborate to regulate the function of Foxp3. The N-terminal domain of Foxp3 facilitates interactions with various molecules, including Foxp1, Eos, Aml1/Runx1, Nfat, Rorα, Tip60, and Hdac7, all of which play roles in different aspects of Treg function and development [21,22,23,24,25,26,27]. Foxp3 expression is further regulated by four distinct conserved non-coding sequences (CNS0-CNS3), which functions as enhancers. CNS2 harbors binding sites for various transcription factors, including Nfat, c-Rel, Runx1, Ets-1, Gata3, and Stat3. These binding sites collectively contribute to the stable expression of Foxp3. The genetic deletion of specific CNS elements reveals distinct roles; some are involved in Foxp3 induction during Treg cell development, while others are crucial for maintaining Foxp3 after lineage commitment. The non-overlapping functions of these CNS elements highlight their individual contributions to the regulation of Foxp3 expression.

Moreover, the transcriptional programs underlying Treg cell function seem to depend on stable and continuous Foxp3 expression, as attenuating Foxp3 expression or deleting the *Foxp3* gene in mature murine Tregs results in a loss of their immunosuppressive function. The loss of Foxp3 in Tregs causes the pathogenesis of autoimmune diseases, while the loss of Foxp3 in the tumor microenvironment (TME) decreases different types of tumor burden due to the upregulation of effector cells. Therefore, Tregs function as a double-edged sword with a protective and pathological role [28]. However, a recent work has shown that tumoral Tregs are particularly sensitive to Foxp3 degradation, which weakens their suppressive function and leads to tumor reduction without major adverse effects [29], providing strong rational for Treg Foxp3 targeting in anti-tumor immunotherapy. Understanding the epigenetic regulation of Tregs is crucial not only for gaining insights into the basic biology of these cells but also for potential therapeutic interventions. Manipulating Tregs’ function through targeted epigenetic modifications is an area of active research with the aim of developing treatments for cancers. This review aims to provide a comprehensive and up-to-date exploration of Treg epigenetics, offering insights that bridge fundamental research with potential therapeutic strategies for cancers.

## 2. Epigenetic Regulations in Treg Cell Development and Maintenance

**Figure 2:**
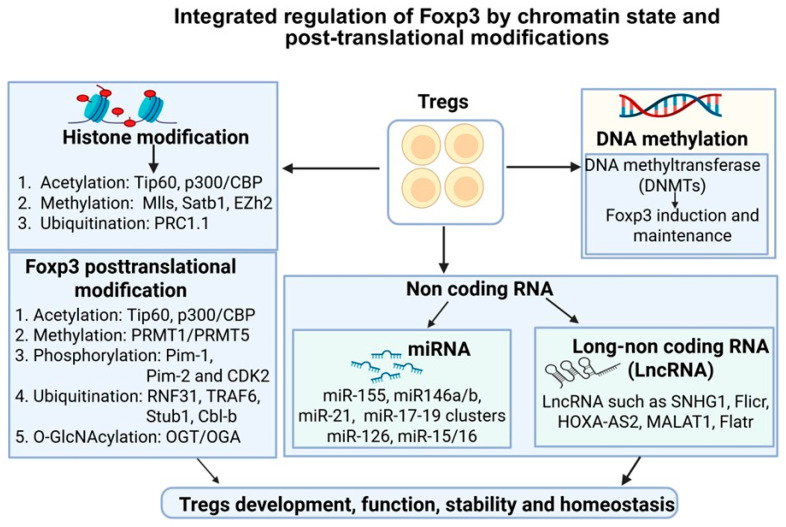
An overview of integrated regulation of Foxp3 by epigenetics and post-translational modifications. Epigenetic processes including DNA methylation, histone modification and non-coding RNAs regulate gene transcription and expression within Tregs. Tregs are also regulated by Foxp3 post-translational modification. Various enzymes involved in different modifications are shown. These modifications regulate Tregs differentiation, homeostasis, activity, stability, and functions.

Epigenetics refers to heritable and reversible molecular mechanisms in gene expression regulation without altering the underlying DNA sequence. The major epigenetic mechanisms include DNA methylation, post-translational histone modifications, and the regulatory actions of non-coding RNAs (ncRNAs) (Figure 2). These processes shape cellular identity and enable dynamic responses to environmental cues. In the immune system, epigenetic regulation is essential for directing the development, differentiation, and functional adaptation of diverse immune cell lineages. During immune cell development, epigenetic mechanisms establish lineage-specific transcriptional programs that guide the formation of specialized immune subsets. Upon activation, immune cells rely on rapid and coordinated epigenetic remodeling to induce effector functions. Notably, epigenetic regulation plays a critical role in the development, stability, and function of Tregs by integrating environmental signals with transcriptional control. Together, these regulatory layers highlight epigenetic pathways as promising therapeutic targets for treating diseases, including cancer.

### 2.1. DNA Methylation

DNA methylation is a biochemical process that entails the conversion of cytosine to 5-methylcytosine, which is facilitated by DNA methyltransferases (DNMTs) such as DNMT1, DNMT3A, and DNMT3B, utilizing S-adenosylmethionine as a substrate. This methylation process takes place during de novo methylation or cell replication, predominantly at CpG islands, resulting in gene silencing [30,31]. Conversely, the Ten-Eleven Translocation (TET) enzymes play a crucial role in demethylation, which is the reversal of DNA methylation and influences gene expression [32,33,34]. DNA methylation provides a heritable epigenetic mechanism maintaining Treg lineage stability. Previous studies have shown that DNA methylation plays a critical role in regulating Foxp3 locus expression, which is demonstrated by differences in DNA methylation between Treg and conventional T cells [35,36]. This was further supported by the inhibitors of DNA methyltransferases that enhance Foxp3 expression with increased Treg cell numbers. The DNA methylation status at Foxp3 gene enhancers, specifically CNSs: CNS0-CNS3, is crucial for both Foxp3 induction and maintenance [37,38]. In another study, the proteins B-lymphocyte-induced maturation protein 1 (Blimp1) and methyl-CpG binding domain protein (MBD)2 were found to promote CNS2 methylation, contributing to Treg cell stability and function [39,40]. Over 100 differentially methylated regions distinguish Tregs from effector T cells, impacting genes like *Foxp3*, *Ctla4*, *Tnfrsf18*, and *Ikzf2*, which are crucial for Treg function and stability [41,42]. The hypomethylation of key loci such as Foxp3 in natural Tregs is a dynamic process that underpins their development. Notably, the loss of Dnmt1 permits T-cell receptor (TCR) stimulation alone to induce Foxp3 expression in precursor cells [43].

It is increasingly evident that active DNA demethylation is essential for both Treg lineage commitment and functional stability [36,37,44,45,46]. The Treg-specific demethylated region (TSDR) is a highly conserved element within the CNS2 locus of the *Foxp3* gene [36,47,48]. nTregs exhibit a stable regulatory phenotype characterized by full demethylation of the TSDR, ensuring long-term immunosuppressive function [49,50]. In contrast, pTregs show partial TSDR demethylation, potentially making them less stable and more susceptible to conversion into effector T cells under certain conditions [50]. Further work showed that in vitro induced Tregs exhibit incomplete demethylation of the TSDR, explaining their transient and less stable regulatory nature [36]. Interestingly, the TSDR becomes remethylated when Foxp3 expression is lost, underscoring its essential role in maintaining Treg stability through epigenetic regulation. The CNS0 region of the *Foxp3* locus interacts with sequence binding protein 1 (Satb1), guiding Foxp3 induction and promoting DNA demethylation within TSDR. Following Treg lineage commitment, the CNS1 and CNS2 enhancers at the *Foxp3* locus undergo coordinated demethylation; however, CNS1 is required primarily for the initial induction of Tregs, which is a stage that precedes the establishment of stable DNA demethylation [51,52,53]. These results suggest that TSDR functions as an epigenetic regulatory element that stabilizes Foxp3 expression and maintains Treg lineage commitment.

Likewise, active DNA demethylation via Ten-Eleven Translocation (Tet) enzymes stabilizes Foxp3 expression through CNS2, controlling multiple gene expressions [32,35]. Additional studies reported that Tet1 and Tet2 are recruited to *Foxp3* in response to TGF-β and IL-2 signaling, supporting the active demethylation and maintenance of Treg-cell-associated immune homeostasis. Moreover, the loss of Tet1 and Tet2 results in a hypermethylation of the *Foxp3* locus, leading to defective Treg cell differentiation and impaired suppressive function [54,55,56]. The demethylation of CNS2 in the *Foxp3* gene facilitates the binding of transcription factors like Runx1-Cbfβ and Foxp3 itself, which in turn enhances Foxp3 stability [57,58]. Another *Foxp3* locus, CNS3, impacts the nTregs and pTregs numbers. This region binds with c-Rel, which is a transcription factor that is essential for *Foxp3* induction. The cooperation between CNS0 and c-Rel-CNS2-CNS3 aids in recruiting transcription factors that support stable demethylation of CNS2 in nTregs [37]. Understanding DNA methylation in Tregs is crucial for unraveling the epigenetic intricacies that govern Treg development, stability, and immune regulatory functions. Collectively, these findings indicate that stable Foxp3 expression depends on DNA demethylation, and epigenetic interference can destabilize Tregs.

### 2.2. Histone Modifications

Histones are a group of highly alkaline proteins found in the cell nucleus around which DNA is wrapped to form chromatin. They play a crucial role in the packaging and regulation of DNA. Histone modifications are chemical changes to histone proteins that significantly influences gene expression by altering chromatin accessibility and structure [59,60]. These modifications, which include acetylation, methylation, phosphorylation, and ubiquitination, are often reversible, allowing cells to dynamically regulate gene expression in response to environmental, cellular, or signaling cues [60,61]. The reversibility of histone modifications is crucial for maintaining flexibility in gene regulation. Different types of histone modification essential for Treg development, function and stability are discussed here.

#### 2.2.1. Histone Acetylation

Histone acetylation is an extensively studied histone modification that profoundly influences transcriptional regulation. It involves the addition of acetyl groups to lysine residues, neutralizing their positive charge. This modification results in a more open chromatin structure, facilitating DNA binding and promoting transcription. Conversely, histone deacetylation, the removal of acetyl groups, is associated with condensed chromatin and leads to transcriptional repression. Histone acetylation is mediated by histone acetyltransferases (HATs) and removed by histone deacetylases (HDACs) [62,63].

Acetylation at the *Foxp3* promoter is essential for initiating Foxp3 transcription [64]. Specific HATs like TIP60 and p300/CBP families regulate Foxp3 acetylation, which is a modification that is crucial for Tregs development and function [25,65,66]. Acetylation also stabilizes Foxp3, improving its regulatory activity, and H3K27ac deposition at the *Foxp3* promoter occurs exclusively in Treg cells [67]. In contrast, HDACs negatively impact Foxp3 expression and Treg function, while HDAC inhibitors enhance Foxp3 levels and enhance their suppression ability [68,69]. Multiple HDACs contribute to the epigenetic regulation of Tregs by modulating chromatin accessibility at the *Foxp3* locus, where deacetylation generally represses transcription and reduces Foxp3 stability. For example, HDAC9 knockout increases histone acetylation at the *Foxp3* promoter and enhances Treg suppressive activity by elevating Foxp3 acetylation [70], whereas HDAC6 knockout results in hyperacetylated Foxp3, stabilizes Treg lineage commitment, and improves suppressive function [71]. In contrast, HDAC3 deletion in Tregs disrupts the epigenetic architecture of the *Foxp3* CNS2 region, impairs Treg metabolic programming, and leads to Treg instability and spontaneous inflammation [68]. Collectively, these findings demonstrate that histone acetylation is a key epigenetic mechanism controlling the generation and function of Treg cells.

#### 2.2.2. Histone Methylation

Histone methylation is catalyzed by histone methyltransferases: protein arginine methyltransferase (PRMT) on arginine residues and lysine methyltransferase (KMT) on lysine residues. Arginine methylation generally promotes transcriptional activation, whereas lysine methylation can either activate or repress transcription depending on the specific lysine residue that is modified. Unlike acetylation, methylation does not alter the histone charge or directly affect histone–DNA interactions, which allows methylation to exert its more diverse and flexible effects on gene regulation. Although once considered irreversible, histone methylation is now recognized as a dynamic and reversible process. Specific methylation marks like H3K4me3 and H3K36me3 associate with active genes, while H3K27me3 and H3K9me3 associate with silenced genes. CNS0 is regulated by histone methyltransferases such as mixed-lineage leukemia (Mll)4 and special AT-rich Satb1 and is required for efficient *Foxp3* induction in response to IL-2 signaling [72,73,74]. Like CNS0, CNS3 undergoes a poised state through H3K4me1 during hematopoiesis. This poised state facilitates the generation of Treg cells by enhancing the sensitivity of precursor cells to TCR signal strength in the presence of other induction signal. H3K4me3 accumulation is prevalent in the promoters of TSS clusters [75]. Meanwhile, the specific enrichment of H3K27me3 is found at Foxp3-bound sites in activated Tregs, contributing to Foxp3-mediated repressive chromatin, particularly under inflammatory conditions. Lysine methyltransferase such as enhancer of zeste homolog 2 (EZH2), which catalyzes H3K27 trimethylation, and MLL1, which mediates H3K4 mono-, di-, and trimethylation, shape Foxp3 expression and Treg stability. In Tregs, the conditional deletion of *Ezh2* leads to the derepression of normally silenced target genes and the destabilization of *Foxp3* transcriptional programs, and it also triggers autoimmunity; conversely, elevated levels of EZH2 in TI-Tregs rely on EZH2 activity for fitness and suppressive function [76,77]. Similarly, the T-cell-specific loss of MLL1 results in spontaneous lymphoproliferation, and its Treg-specific deletion causes systemic autoimmunity due to impaired Treg function [78]. In addition to methyltransferases, lysine marks are removed by demethylases such as lysine demethylase 1/lysine-specific demethylase 1 (KDM1/LSD1) and Jumonji C (JmjC) [79,80]. Functionally, the pharmacological inhibition of LSD1 leads to the production of IL2 and IFN-γ production, impairs Tregs function and enhances anti-tumor immunity [81]. Methylated histones recruit proteins like methyl-CPG-binding domain proteins such as Mbd1, which in turn assemble chromatin remodeling and histone-modifying complexes to reinforce DNA methylation and transcriptional repression. Recent studies suggest that Mbd2 promotes the demethylation of *Foxp3* [40], potentially impacting Treg numbers or functional stability. More work is still needed to fully understand how histone methylation shapes Foxp3 regulation in Tregs.

#### 2.2.3. Histone Ubiquitination

Ubiquitination centers on two monoubiquitylation marks, H2A-K119ub1 and H2B-K120ub1, that tune nucleosome dynamics and regulate transcription [82]. In Tregs, non-canonical polycomb repressive complexes (PRC1.1) writes H2A-K119ub1 at active promoters licensing rapid, tissue-context gene responses that bolster functional Treg stability [83]. This is counter-balanced by the polycomb repressive deubiquitinase complex (BAP1 with ASXL1/2/3, FOXK1/2), which removes H2AK119ub1 to fine-tune polycomb output and permit gene activation [84]. A CRISPR screen extends to the H2B axis: USP22, a SAGA deubiquitinase (DUB), acts as a positive Foxp3 regulator, while RNF20 (H2B E3) is a negative regulator [85]. Consistently, the Treg-specific loss of Usp22 increases the levels of H2A-K119ub1 and H2B-K120ub1 at the *Foxp3* locus correlating with reduced Foxp3 expression, spontaneous autoimmunity and enhanced anti-tumor immunity [85]. Overall, these findings underscore ubiquitination as a key mechanism in regulating Treg stability.

#### 2.2.4. Histone Phosphorylation

Histone phosphorylation plays a pivotal role in chromatin remodeling including transcription, DNA replication, chromosome segregation, DNA damage, and apoptotic responses. Unlike acetylation and methylation, histone phosphorylation establishes an interaction between other histone modifications and provides a platform for effector proteins, triggering downstream events. This modification is crucial for chromosome condensation during cell division, transcriptional regulation, and DNA damage responses [86,87,88,89,90]. All four core histones’ phosphorylation and dephosphorylation are regulated by various protein kinases and phosphatase enzymes that have emerged as a mechanism for the regulation of diverse chromatin-related events. To date, analyses of histone phosphorylation in Treg cells are limited.

### 2.3. Non-Coding RNA

A non-coding RNA (ncRNA) is a functional RNA molecule that does not undergo translation into a protein [91,92]. Despite not encoding proteins, ncRNAs carry important biological information and encompass various types, including microRNAs and long non-coding RNAs (lncRNAs). Table 1 summarizes key miRNAs, their reported functions, validated or proposed target genes, the associated regulatory T cell phenotypes, and the species.

#### 2.3.1. MicroRNAs

MicroRNAs (MiRNAs) commonly consist of ~22 nucleotides, and they function in RNA silencing and the post-transcriptional regulation of gene expression. Their altered expression is observed in biological and pathological processes. MiRNAs bind to their specific target RNAs in either the translational regions or the 3′ untranslated regions (3′UTR). Several of the following miRNAs (Table 1) play essential roles in the regulation of Tregs’ development, stability, homeostasis, and function [93,94,95].

(1) MiR-155: It is highly expressed in Tregs, and its expression is regulated by Foxp3 and required for Treg development. The *miR-155*-deficient mice had fewer Tregs in thymi and spleens; however, their suppressive function in vitro remains unaffected [96]. This miRNA directly targets suppressor cytokine signaling 1 (*Socs1*), which suppresses IL-2 signaling [97]. In parallel with *miR-155*-deficient mice, *Socs1* transgenic mice exhibited a reduced number of Tregs, indicating that *miR-155* may regulate Treg proliferation by targeting IL-2 signaling through the inhibition of SOCS1 expression.

(2) MiR-146a/b: *miR-146* consists of two members, *miR-146a* and *miR-146b*, which are located in different chromosomes. *miR-146a*, but not *miR-146b*, was highly up-regulated in Treg cells. *miR-146a* deficiency results in increased numbers but an impaired function of Treg cells [98]. Further, *miR-146a* deficiency leads to an increase in peripheral Tregs and results in lymphoproliferative and myeloproliferative syndrome in mice at 6 months of age. *miR-146* ensures a Treg-cell mediated control of Th1 responses through targeting the key transcription factor stat1.

(3) MiR-21: miR-21 is widely upregulated in multiple solid tumors and hematological malignancies and is induced by hypoxia, where it promotes oncogenesis by targeting tumor-suppressor genes such as such as *Pdcd4* (programmed cell death protein 4) and *Reck* (reversion-inducing cysteine-rich protein with kazal) [99]. In the immune cells, miR-21 is expressed at higher levels in Tregs compared with conventional T cells [100,101]. However, genetic deletion studies demonstrate that *miR-21* is dispensable for both induced and natural Treg differentiation, as *miR-21*^−/−^ mice exhibit unaltered Foxp3 expression and normal Treg development [101]. While Treg-specific *miR-21* deficiency does not impair suppressive function at steady state, it alters cytokine production under inflammatory conditions, including increased IL-17 and IL-10 via modulation of the IL10/STAT3 activity [102]. Collectively, these findings indicate that *miR-21* functions as a context-dependent modulator of Treg activity rather than an essential regulator of iTreg differentiation.

(4) MiR-17-92: It consists of six mature microRNAs, including *miR-17*, *miR-18a*, *miR-19a*, *miR-20a*, *miR-19b*, and *miR-92*. miR-17-92 inhibits iTregs differentiation. Eliminating the *miR-17-92* cluster in CD4^+^ T cells promotes Foxp3 expression [103]. The *miR-17-92*-deficient Tregs show no signs of disease in mice, suggesting that *miR-17-92* in Tregs is dispensable for Tregs under healthy conditions [103]. However, *miR-17-92*-deficient Tregs in an acute experimental autoimmune encephalomyelitis (EAE) mice model showed a decrease in the number of antigen-specific Tregs and the decreased secretion of immunosuppressive cytokine IL-10. Therefore, *miR-17-92* plays an important role in the inflammatory condition to regulate antigen-specific Tregs and immunosuppressive function. PTEN is the key target of *miR-19b*, while *miR-17* acts through TGFβRII and the newly identified target CREB1. Loss of the *miR-17-92* cluster in CD4 T cells disrupts the balance between Th1 cells and inducible Tregs, enabling tumor immune evasion [103].

(5) MiR-126: *miR-126* is highly expressed in Tregs. The in vivo abrogation of *miR-126* dramatically downregulates the expression of Foxp3, CTLA4, and GITR in Tregs and impairs suppressive function [104]. *miR-126* directly targets the 3′UTR of p85β, which is an important regulatory subunit of PI3K involved in PI3K/Akt pathways [105,106]. These results suggest that *miR-126* alters Foxp3 expression and impairs suppressive function through the PI3K/AKT pathway in Treg.

(6) MiR-15/16: The *miR-15/16* family consists of abundant miRNAs that are in two separate clusters (*miR-15a/miR-16-1* and *miR-15b/miR-16-2*). *miR-15b/16* is more abundant in Tregs [107] and regulates T effector responses [108,109], Treg differentiation [107] and eTreg response [110]. *miR-15/16* acts as a tumor suppressor [111]. The loss of *miR-15/16* increases effector Tregs and their suppressive function by derepressing Irf4 and directly targeting neuritin, an Irf4-dependent molecule [110], while overexpression reduces Foxp3 and CTLA4 [112]. In cancer, miR-15/16 inhibits proliferation and induces apoptosis by targeting oncogenic or pro-survival genes BCL2, CCND1, MCL1, and WNT3A [111].

These miRNAs serve as key regulators in Treg biology, influencing their development, stability, and suppressive function.

#### 2.3.2. Long Non-Coding RNA

Long non-coding RNAs (lncRNAs) are mRNA-like transcripts longer than 200 nucleotides that lack significant protein-coding capability. Importantly, IncRNAs play a crucial role in modulating chromatin structure and regulate gene expression at transcriptional and post-transcriptional levels. However, lncRNAs’ characteristics and their function and mechanisms in Tregs still remain obscure [113,114,115]. There are a few studies related to cell signaling regulation via lncRNAs in Treg differentiation (Table 1).

(1) The lncRNA Snhg1(small nucleolar RNA host gene 1): It promotes Tregs differentiation and contributes to the progression of multiple cancers [116]. It interacts with miR-448 and then prevents the inhibition of indoleamine 2,3-dioxygenase (IDO), which is a key enzyme that is required for Tregs expansion. Consistent with this, SNHG1 knockdown reduces Tregs activation and limits tumor progression, indicating that SNHG1 supports immune evasion through Treg-mediated suppression [116,117].

(2) LncRNA Flicr (Foxp3 long intergenic noncoding RNA): The lncRNA Flicr is located adjacent to Foxp3, which is expressed specifically in mature Tregs and is a negative regulator of Foxp3 expression [118]. It impairs Tregs differentiation and stability through decreasing the chromatin accessibility in the Foxp3 locus.

(3) The lncRNA Malat1 (metastasis-associated lung adenocarcinoma transcript 1: LncRNA MALAT1 is involved in the development and progression of various cancers [119,120]. In the immune system, Malat1 has been studied in different immune cell types, including Tregs [121,122,123,124]. MALAT1 promotes immune tolerance by driving the development of tolerogenic dendritic cells and Tregs through the miR-155/dendritic cell-specific intercellular adhesion molecule 3-grabbing nonintegrin/IL10 signaling axis [124]. In the EAE mouse model, Malat1 expression is downregulated, leading to reduced CD4 expression and a corresponding decrease in Treg differentiation [123]. Furthermore, the siRNA-mediated downregulation of MALAT1 skews T-cell differentiation toward a Th1/Th17 cell profile while suppressing Treg differentiation, suggesting a potential anti-inflammatory effect for MALAT1 [123]. 

(4) The lncRNA Flatr (Foxp3-specific lncRNA anticipatory of Tregs): It is highly conserved and enriched in activated Tregs, where it supports Treg development by upregulating Foxp3 expression [125]. Mice with the Treg-specific deletion of *Flatr* show delayed Treg induction, highlighting its important role in controlling Foxp3 expression.

(5) The IncRNA HOXA-AS2 (Homeobox A Antisense RNA 2): The upregulation of lncRNA *HOXA-AS2* in glioma has been reported [126]. It is located in the *HOXA* gene cluster and promotes Treg cell proliferation and immune tolerance through the inhibition of *miR-302a* and activation of the KDM2A/JAG1 axis [126].

(6) Other IncRNAs: IncRNAs *MAFTRR* (MAF Transcriptional Regulator RNA), also known as *Linc-MAF4*, *TH2-LCR* (T Helper 2 Locus Control Region), and *IFNG-AS1* (Interferon Gamma Antisense RNA 1) are among the several Treg-associated lncRNAs found to be upregulated in breast cancer and lung cancer [127,128]. *MAFTRR* is a chromatin-associated Th1-specific lncRNA that regulates the differentiation of these cells through suppressing MAF expression in the activated CD4^ +^ T cells [129,130]. *TH2-LCR* has an indispensable role in the regulation of Th2 cytokine genes [131]. *IFNG-AS1* is a positive regulator of IFN-γ in T and NK cells, and IFN-γ-associated chemokines have been found to be possible biomarkers of metastasis and prognosis in cancer [132,133].

Understanding the roles and mechanisms of these lncRNAs offers a deeper insight into the complex regulatory networks governing immune responses, immune tolerance, and the balance between inflammatory and suppressive activities within the immune system.

**Table 1:** List of miRNAs and LncRNAs, their impact on Tregs with their targets, and the species involved.

Regulators	Impact on Tregs	Targets	Species	References
Non-coding RNAs				
(i) miRNAs				
miR-155	Maintains suppressive function Treg development	SOCS1	Mice	[96,97]
miR-146a/b	Promote suppressive function	STAT1	Mice	[98]
miR-21	Dispensable in iTreg cell differentiation	IL-10/STAT3	Mice	[102]
miR-17-92	Promote Tregs differentiation	Pten/TGFbRII/CREB1	Mice	[103]
miR-126	Maintains suppressive function	PI3K/Akt pathway	Mice, Human	[104]
miR-15/16	eTreg differentiation and function	IRF4/Neuritin	Mice	[110]
(ii) LncRNA				
Snhg1	Suppression of Treg differentiation	miR-448/IDO	Human	[116]
Flicr	Negative regulator of Foxp3	Foxp3	Mice, human	[118]
Malat1	Increasing T cells	miR-155/DC-SIGH/IL10 axis	Mice	[123,124]
Flatr	Treg induction	Foxp3	Mice	[125]
Hoxa-AS2	Treg cell proliferation	KDM2A/JAG1 or miR-302a	Mice, Human	[126]
MAFTRR	Treg-associated lncRNAs	MAF	Human	[129,130]
TH2-LCR	Treg-associated lncRNAs	Th2 cytokine	Mice	[131]
IFNG-AS1	Treg-associated lncRNAs	IFN-y	Human	[132,133]

### 2.4. Chromatin Organization and Accessibility in Tregs

#### 2.4.1. Super-Enhancer Reprogramming in Tregs

Super-enhancers (SEs) are highly active cis-regulatory regions in the genome that contain dense assemblies of transcription factors, cofactors, and chromatin regulators to drive robust gene expression programs that define cell identity, promote tumorigenesis, and enhance cellular aggressiveness [134,135]. In Tregs, lineage identity is tightly regulated by a specialized SEs landscape that establishes and stabilizes the expression of core Treg signature genes, including *Foxp3*, *Il2ra*, *Ctla4*, *Tnfrsf18*, and *Ikzf2* [136]. These SEs are characterized by open and DNA-demethylated chromatin, a strong enrichment of H3K27ac, H3K4me3, and H3K4me1, and the depletion of the H3K27me3 mark [137]. Notably, SEs activation begins early during nTreg differentiation, preceding detectable transcription, indicating that the formation of SEs is an instructive event that drives lineage commitment rather than a consequence of transcription [138]. At the *Foxp3* locus, the CNS0 SE functions as a critical regulatory hub bound by the pioneer factor Satb1 with cooperative STAT5 signaling initiating *Foxp3* transcription and stabilizing Treg fate [72,139]. SEs drive cohesin-dependent chromatin looping to their target promoters, thereby amplifying transcriptional output. The disruption of key SEs, particularly CNS0, severely impairs nTreg development, highlighting their essential role in Treg lineage specification [140].

Within the TME, TI-Tregs retain this core SE framework to preserve lineage stability but undergo extensive SE reprogramming in response to local cues [141,142]. Compared to pTregs, TI-Tregs acquire newly formed or amplified SEs at genes encoding immunosuppressive molecules, leading to increased suppressive function and stability [143,144]. This tumor-specific SE landscape is shaped by chronic TCR stimulation and tumor-derived signals such as IL-2, TGF-β, and hypoxia, and it is reinforced by Foxp3 together with environment-responsive transcription factors including BATF, IRF4, and STAT5 [145,146]. Adaptive SE reprogramming enhances the suppressive potency, persistence, and functional specialization of TI-Tregs, enabling them to effectively dominate anti-tumor immune responses while maintaining a stable Treg identity. Importantly, the disruption of SE-associated transcriptional machinery, such as the inhibition of BRD4 or CDK7, selectively impairs TI-Treg function, highlighting SE reprogramming as a potential therapeutic vulnerability in cancer immunotherapy [147,148,149].

#### 2.4.2. Nucleosome Positioning and Chromatin Accessibility in Tregs

Beyond DNA methylation and histone modifications, nucleosome positioning constitutes a critical yet comparatively less characterized layer of epigenetic regulation in Tregs that directly influences transcriptional control. Recent advances in chromatin accessibility profiling, most notably ATAC-seq, now enable high-resolution mapping that simultaneously access chromatin accessibility and nucleosome organization in Tregs [147,150,151]. These studies demonstrate that accessible regions correlate with Treg-specific transcription factor occupancy and promoter accessibility, showing that ATAC seq can characterize the open chromatin landscape, which is important for Treg suppressive and effector functions. Within the TME, Tregs exhibit a distinct chromatin accessibility profile relative to peripheral Tregs [152,153], reflecting differences in accessible loci linked to the function and phenotype in tumors. Collectively, these findings indicate that Tregs maintain a unique chromatin and nucleosome landscape that underlies their lineage identity and regulatory functions in both peripheral tissues and tumors.

#### 2.4.3. Metabolic–Epigenetic Integration in Tregs

Metabolic and epigenetic pathways in Tregs are tightly interconnected, as metabolic intermediates function not only as fuels for ATP production but also serve as essential substrates and cofactors for enzymes that modify the epigenome, thereby regulating Treg differentiation, function, stability, and plasticity [154,155]. This metabolic–epigenetic crosstalk is further shaped by hypoxia, a hallmark of inflamed tissues and TME, which profoundly influences Tregs fate and function [156]. Tregs activation and lineage commitment are accompanied by metabolic reprogramming, underscoring the dual role of cellular metabolism in bioenergetic support and epigenetic regulation [152,155,157]. Beyond energy generation, metabolic pathways supply key metabolites that directly regulate the chromatin-modifying enzymes controlling Tregs development [158]. Under hypoxic conditions, the stabilization of hypoxia-inducible factor-1α (HIF-1α) promotes glycolysis, enhancing glucose- and glutamine-dependent O-GlcNAcylation that stabilizes Foxp3 expression [159,160]. Increased glycolytic flux also elevates lactate production, which inhibits HDAC activity [155] and supports the suppressive function and proliferation of intratumoral Tregs [161]. Mitochondria-derived metabolites, including α-ketoglutarate (α-KG), 2-hydroxyglutarate (2-HG), succinate, and fumarate, regulate DNA and histone demethylases, counterbalancing the activity of HMTs and DNMTs associated with one-carbon metabolism and the methionine cycle [162,163]. Histone acetylation broadly depends on acetyl-CoA availability from multiple metabolic pathways, while butyrate, a product of bacterial anaerobic fermentation, acts as an HDAC inhibitor to increase histone H3 acetylation at the Foxp3 locus and enhance Treg cell function [155]. Finally, the SIRT family of deacetylases requires NAD^+^ as a cofactor, linking hypoxia-driven alterations in the NAD^+^/NADH ratio to epigenetic regulation in Treg cells [164,165]. Collectively, these signals coordinate Treg differentiation, stability and function by regulating chromatin modification and the transcriptional program.

## 3. Foxp3 Post-Translational Modification Required for Treg Stability

Foxp3, a master regulator of Tregs, is tightly regulated by multiple post-translational modifications (PTMs) such as acetylation, methylation, phosphorylation, ubiquitylation, and O-GlcNAcylation, which are essential for Treg stability, and suppressive function (Figure 2, Table 2).

Foxp3 acetylation is driven by HATs: notably, the Tip60 and p300/CBP families govern Foxp3 acetylation, which is key for Treg cell development and function and lineage stability [65,66,166]. The Foxp3 N-terminal region (aa106–190) associates with Tip60 and HDAC7 to form a repressive complex that inhibits IL-2 transcription in T cells [25]. The Treg-specific loss of Tip60 triggers early, scurfy-like lethal autoimmunity, underscoring its non-redundant role [166,167]. In contrast, p300 acetylates Foxp3 at multiples sites (K31, K262, and K267), which prevents proteasome-mediated degradation, raising Foxp3 protein levels and strengthening the Foxp3-dependent repression of IL-2 [65,168]. On the other hand, the acetylation of Foxp3 by p300 at K250 and K252 relaxes Foxp3 dimers and reduces its suppressive function [169]. When both P300/Tip60 are present, Foxp3 is acetylated at K179 and K227, whereas the loss of either enzyme results in weak Foxp3 acetylation [167,170,171]. CBP, a paralog of p300, is likewise important in certain inflammatory or lymphopenic settings, and the combined deletion of CBP and p300 in Tregs causes fatal autoimmunity within one month of age [65,172], revealing partial redundancy within the p300/CBP pair. Conversely, deacetylases like SIRT1 and HDACs (HDAC1/2, HDAC3 HDAC6/7, HDAC9/11) reduce Foxp3 stability and activity [65,68,69,173,174,175,176]. However, blocking these deacetylases boosts Foxp3 acetylation and enhances Treg functions. Taken together, these Foxp3 modifications support Treg lineage stability and suppressive function.

Foxp3 Arginine methylation is carried by arginine methyltransferase PRMT1 and PRMT5. PRMT1 methylates arginine residues on R48 and R51 [166,170]. The inhibition of this methylation results in an attenuation of suppressive activity. PRMT5 is highly expressed in a variety of cancers, and several inhibitors have been developed that target PRMT5 for cancer therapy [177]. PRMT5 preferentially binds to Foxp3 and catalyzes the methylation on R27, R51 and R146 of Foxp3, and a conditional deletion of *Prmt5* gene in Tregs leads to scurfy-like autoimmune diseases [170,178]. Collectively, PRMT5 stabilizes Foxp3 and enhances its transcriptional activity [166], thereby maintaining Treg’s lineage identity and suppressive capacity. Both PRMT1 and PRMT5 regulate Foxp3 function and plasticity.

Foxp3 phosphorylation plays a significant role in regulating Treg function. The Nemo-like kinase (NLK)-mediated phosphorylation of Foxp3 at different sites stabilizes protein levels by hindering its proteasomal degradation through ubiquitin-mediated pathways [170,179]. Cyclin-dependent kinase 2 (CDK2) also impacts Treg function by phosphorylating Foxp3 at S19 and T175, thereby negatively influencing its stability and activity [180]. Mutations in the serine and threonine residues targeted by CDK2 result in an increased Foxp3 half-life and improved function, as observed in CDK2-deficient Tregs, which exhibit heightened suppressive abilities. Both Pim-1 and Pim-2 kinases negatively regulate Tregs’ suppressive function by phosphorylating Foxp3 [181,182]. Pim-2 kinase phosphorylates multiple sites in the Foxp3 N-terminal domain. Foxp3 is highly phosphorylated at S418 with dephosphorylation at this residue mediated by the phosphatase PP1 [181]. Phosphorylation at S422 by the kinase Pim-1 negatively impacts Foxp3 activity, which is countered by the opposing action of the PP1 phosphatase.

Foxp3 ubiquitination shapes Foxp3 fate in Tregs. Under inflammatory or stress conditions, K48-linked ubiquitin chains promote Foxp3 proteasomal degradation, while K63-linked chains tend to stabilize Foxp3 and support its nuclear localization. Among the E3 ubiquitin ligases, STIP1 homology and U-box-containing protein 1 (Stub1) and Casitas B-lineage lymphoma proto-oncogene-b (Cbl-b) drive degradative ubiquitination and weaken Treg function; meanwhile, TNF Receptor-Associated Factor 6 (Traf6) and RING Finger Protein 31 (Rnf31) enhance Foxp3 stability and Treg suppression [183,184,185,186,187]. These ligases regulate multiple mono- or poly-ubiquitination Foxp3 at multiple sites (Table 2). In addition, another E3 ligase, hydroxymethylglutaryl-CoA reductase degradation protein 1 (Hrd1), supports Foxp3 stability and boosts Tregs suppressive function mainly by suppressing ER-stress signaling rather than by directly ubiquitinating Foxp3 [188]. On the deubiquitinase side, ubiquitin-specific peptidases (USP7, USP21, USP22, and USP44) have been reported to interact with or deubiquitinate Foxp3, preventing its degradation and influencing Treg homeostasis, stability, and function [85,170,189,190,191,192,193,194,195]. USP7 preserves Foxp3 homeostasis while USP21 prevents Foxp3 degradation by deubiquitinating Foxp3 at multiple sites (Table 2). Overall, the balance between Foxp3-targeting E3 ligases and deubiquitinases set Foxp3 abundance and activity, thereby controlling Treg function.

Foxp3 O-GlcNAcylation is added by O-GlcNAc transferase (OGT) and removed by GlcNAcase (OGA), which is a nutrient sensing PTM that links the hexosamine pathway to Treg function [196]. Foxp3 can be modified at multiple sites and promotes protein stability and sustains suppressive function, especially under metabolic stress [170,197] (Table 2). The Treg-specific ablation of OGT causes severe autoimmune syndrome in mice due to Treg lineage instability and loss of effector-Treg populations [197]. Mechanistically, O-GlcNAcylation may counteract ubiquitination to stabilize Foxp3 protein, and a loss of O-GlcNAcylation destabilizes the Foxp3 protein. Collectively, these PTMs form an integrated regulatory network that preserves Foxp3 expression, ensures Treg functional stability, and allows adaptive responses in inflammatory microenvironments.

**Table 2:** Post-translational modification of Foxp3 with location, modifiers, impact on Tregs and their functions.

Types of PTM	Location	Modifiers	Impact on Tregs	Function	Human or Mice	References
Foxp3 Acetylation	K31, K262, K267	P300	Positive	Foxp3 stability	Human, Mice	[168]
	K250, K252	P300	Negative	Reduced suppressive function	Human	[169]
	K179, K227	Tip60/ p300	Positive	Foxp3 stability	Human	[171]
Foxp3 Methylation	R48, R51	PRMT1	Positive	Foxp3 function and plasticity	Human	[166,170]
	R27, R51, R146	PRMT5	Positive	Foxp3 function and plasticity	Human	[170,178]
Foxp3 Phosphorylation	S19, S156, S189, S273, S278, S295, T341	NLK	Positive	Foxp3 stability	Human, Mice	[179]
	S19, T175	CDK2	Negative	Foxp3 stability	Mice	[180]
	S422	Pim-1	Negative	DNA binding activity	Human	[182]
	S33, S41	Pim-2	Negative	Protein binding activity	Mice	[181]
	S418	PP1	Positive	Foxp3 function	Human	[181]
Foxp3 Ubiquitination	K227, K250, K263, K268	Stub1	Negative	Foxp3 degradation	Human	[184]
	K31, K200, K250, K263, K268, K382, K393, K416	RNF31	Positive	Improved Foxp3 protein level and enhanced suppressive functions	Human	[187]
	K262	TRAF6	Positive	Foxp3 localization	Mice	[186]
	K249, K251, K263, K267, K393	USP7 DUB	Positive	Foxp3 homeostasis	Mice	[195]
	K206, K216, K227, K252, K277, K332, K393	USP21 DUB	Positive	Prevents Foxp3 degradation	Human	[191]
		USP22	Positive	Prevents Foxp3 degradation	Mice	[85,193]
		USP44	Positive	Prevents Foxp3 degradation	Mice	[170,194]
Foxp3 O-GlcNAcylation	T38, S57, S58, S270, S273	OGT /OGA	Positive	Foxp3 stability	Mice	[170,197]

## 4. Cancer Type-Specific Epigenetic Regulation of Tregs

### 4.1. Mechanism of Treg Infiltration and Suppressive Function in Cancer

Cancer remains a major threat to global health with the American Association for Cancer Research predicting that by 2040, the worldwide cancer burden will reach 28 million cases and 16.2 million deaths [198,199]. Tumors develop within a complex TME composed of diverse immune cells population including Tregs, immunosuppressive molecules (e.g., PD1, PDL1, CTLA4), and tumor-associated immune cells like myeloid-derived suppressor cells and tumor-associated macrophages that collectively contribute to immune inhibition [200]. Although immune checkpoint inhibitors targeting these molecules have shown promise in reactivating antitumor immune responses, their therapeutic efficacy depends strongly on factors like immune cell composition within the TME, particularly the presence of CD8 T cells. Therefore, effective cancer immunotherapies will require a deeper understanding of these immune suppressive networks and the development of strategies to modulate them for improved therapeutic outcomes.

While Tregs are essential for maintaining immune homeostasis and preventing autoimmunity, their accumulation within the TME is often associated with poor prognosis across many solid tumors [145]. Compared with peripheral or circulating Tregs, TI-Tregs exhibit heightened immunosuppressive capacity, which is characterized by elevated Foxp3 expression [85,157,201]. Tregs infiltrate the TME through three primary mechanisms including chemotactic recruitment, local differentiation from conventional T cells, and functional adaptation within the tumor milieu [202,203,204,205]. They express chemokine receptors such as CCR4, CCR6, and CCR8, which mediate their migration toward tumor-derived chemokines including CCL17, CCL20, and CCL22. In addition, TGF-β within the TME promotes the differentiation of peripheral Tregs and enhances their suppressive capacity. Once established within tumors, Tregs exert immunosuppressive effects through several intrinsic mechanisms. These include the secretion of inhibitory cytokines (IL-10, TGF-β, IL-35), the expression of immune checkpoint molecules (CTLA4, PD1, LAG3), and metabolic disruption via CD39/CD73-mediated adenosine production, as mentioned by several review articles [206,207,208,209]. The stability and suppressive function in tumors are maintained by three key factors: TCR signaling, metabolic cues (including hypoxia, nutrient deprivation, and lactic acid accumulation), and epigenetic regulation (through chromatin remodeling and histone modification [48,210]. Epigenetic regulators like EZH2 and the TET family, along with ATP-dependent chromatin remodeling complexes including SWI/SNF factor, play crucial roles in maintaining Treg lineage stability and transcriptional programs essential for suppressive function [35,54,55,56,211].

Beyond these Treg-intrinsic mechanisms, tumor-intrinsic epigenetic programs indirectly shape Tregs’ accumulation and activity by establishing an immunosuppressive TME. Tumor cells and associated stromal or myeloid cells secrete soluble factors such as TGF-β and all-trans retinoic acid, which enhance epigenetic accessibility at the *Foxp3* locus and promote the differentiation of conventional T cells into induced Tregs. Cancer cells rely heavily on aerobic glycolysis, resulting in glucose depletion, hypoxia, and lactate accumulation within the TME. These tumor-driven metabolic constraints suppress mTOR signaling and IFN-γ production in tumor-infiltrating lymphocytes, thereby reducing cytotoxic effector function [50,203]. Within this metabolically hostile environment, Tregs preferentially utilize oxidative phosphorylation complex and fatty acid oxidation to maintain suppressive function, enabling them to thrive under hypoxic and nutrient-poor conditions. HIF-1α, activated within TME, further promotes Treg differentiation by upregulating Foxp3 and increasing PDL1 expression, enhancing their ability to suppress effector T-cell function [50,203]. Additionally, tumor-derived metabolites such as lactate and fatty acids substrates also regulate transcription factors essential for Treg identity. Tumor-derived exosomes further contribute to immune reprogramming by transferring regulatory molecules that support Treg induction and function. Moreover, the epigenetic upregulation of immune checkpoint ligands such as PDL1 and the epigenetic control of chemokine expression, including CCL17 and CCL22, by tumor and stromal cells facilitate selective Treg recruitment, collectively creating a permissive niche that sustains Tregs accumulation and function. In this context, the epigenetic regulation of Tregs is vital in cancer because it controls their stability and suppressive function, allowing tumors to evade immune attack. Through mechanisms such as *Foxp3* gene demethylation, histone modifications, and regulation by microRNAs and long non-coding RNAs, Tregs become epigenetically “locked” in an active state that maintains immune tolerance within the TME. This persistent suppression weakens anti-tumor immunity and contributes to resistance to immunotherapies. Targeting these epigenetic pathways such as EZH2, p300, or specific miRNAs offers a promising strategy to reprogram Tregs and restore effective immune responses against cancer.

### 4.2. Melanoma

Melanoma is characterized by exceptional phenotypic plasticity and adaptability, which is driven not only by genetic mutations but also by heritable yet reversible regulatory mechanisms that alter gene expression without changing the DNA sequence [212,213]. Melanoma cells employ multiple immunosuppressive strategies to evade immune surveillance. Through dysregulated non-coding RNA networks together with aberrant histone modifications, chromatin remodeling collectively supports the initiation, metastasis, immune evasion, and therapy resistance in melanoma disease [214]. Multiple tumor intrinsic lncRNAs, including *MALAT1*, BRAF-activated noncoding RNA (*BANCR*), HOX Transcript Antisense Intergenic RNA (*HOTAIR*), and *SNHG5*, are frequently upregulated in melanoma and contribute to tumor growth and progression [215,216,217,218,219]. *MALAT1* promotes melanoma proliferation and metastasis by acting as a competing endogenous RNA (ceRNA) that sponges miR-22, thereby derepressing MMP14 and Snail [217]. The *BANCR*, particularly enriched in BRAF-mutant melanoma, enhances tumor cell proliferation in vitro and in vivo through modulation of the MAPK signaling pathway [219]. *HOTAIR* functions as an epigenetic regulator, which was originally identified for its role in chromatin remodeling and gene repression. It is highly expressed in metastatic melanoma and promotes cell motility and invasion by regulating NF-κB signaling and EMT-associated adhesion molecules, including N-cadherin and E-cadherin [215]. *SNHG5* similarly functions as a ceRNA, sponging *miR-26a-5p* to increase ca influx mediator transient receptor potential canonical 3 (TRPC3) expression, with SNHG5 knockdown suppressing proliferation and inducing apoptosis [216]. Although these lncRNAs primarily drive melanoma progression via tumor-intrinsic oncogenic programs, the pathways they regulate such as extracellular matrix remodeling, oncogenic signaling, and metabolic adaptation are known to shape the tumor immune microenvironment. Through these effects, melanoma-associated lncRNAs may indirectly foster a Treg-permissive, immunosuppressive niche, even though the direct epigenetic regulation of Tregs by lncRNAs in melanoma has not yet been demonstrated.

Tumor-intrinsic epigenetic programs modulate immune checkpoint expression, including PDL1, through chromatin accessibility and the hypoxia-driven activation of HIF1α, reinforcing local immune suppression [220]. Beyond PDL1 expression, melanoma cells upregulate IDO-1, suppressing natural killer cells cytotoxicity, and secrete chemokines that recruit Tregs, which inhibit effector T-cell function through suppressive cytokines [221,222]. Melanoma-derived GM-CSF, IL-6, and exosome-associated microRNAs further promote the recruitment and activation of myeloid-derived suppressor cells (MDSCs) that inhibit effector T cells function [223]. Tumor-secreted factors such as VEGF-A, prostaglandin E2, and IL-10 impair T-cell infiltration and survival by downregulating endothelial adhesion molecules, including ICAM-1, and inducing FasL-mediated CD8^+^ T-cell apoptosis. Epigenetically, melanoma cells suppress effector T-cell recruitment by hypermethylating chemokine promoters such as CXCL9 and CXCL10, thereby limiting directional cues for T-cell trafficking. Genome-wide DNA methylation analyses reveal extensive epigenetic heterogeneity in melanoma, affecting genes involved in epithelial–mesenchymal transition PI3K/mTOR signaling, metabolism and tumor suppression [224,225,226]. Genes involved in cell differentiation, epithelial–mesenchymal transition, are frequently differentially methylated in melanoma [225,226]. Overall, melanoma cells use epigenetically regulated pathways to create an immunosuppressive TME.

In immune cells, *miR-155* is known to be essential for Treg maintenance under homeostatic conditions by repressing SOCS1, thereby sustaining IL2-STAT5 signaling and promoting Treg proliferation including survival [97,227]. In contrast, within the TME, particularly in the B16 melanoma model, *miR-155* adopts a pro-inflammatory, anti-tumor role by promoting effector CD8^+^ T-cell activation and cytokine production, thereby facilitating immune-mediated tumor control rather than supporting Treg-dependent suppression [228,229]. Treg-signature genes such as Ctla4 and Tnfrsf18 exhibit promoter-associated H3K4me3 together with DNA hypomethylation, while effector-lineage genes H3K27me3 modifications [41]. A histone methyltransferase, EZH2, catalyzes H3K27me3 is crucial for Treg identity and function [76]. Treg-specific *Ezh2* deletion destabilizes Tregs and disrupts their suppressive program, resulting in heightened immune activation [76]. Extending this to cancer, EZH2 is required within tumor-infiltrating Tregs (TI-Tregs) to preserve their suppressive identity [77]. The genetic deletion or pharmacologic inhibition of EZH2 in TI-Tregs reprograms them and enhances anti-tumor immunity in melanoma mouse models through the recruitment and activity of CD8^+^ and CD4^+^ effector T cells that eliminate tumors, providing a selective way to target TI-Tregs [77]. In contrast to genetic Foxp3 deficiency that prevents functional Treg development, induced Foxp3 degradation enhances anti-tumor immunity in melanoma with minimal adverse effects [29]. Collectively, these studies show that the epigenetic regulation of Tregs programs its differentiation, stability and suppressive function in melanoma.

### 4.3. Lung Cancer

Lung cancer is the number one cause of cancer and is the second leading cause of cancer-related deaths in the United States, posing a major threat to people’s health worldwide. Several studies have explored Tregs as a potential prognostic biomarker in lung cancer [230]. Tregs influence the TME during the progression of lung cancers, and accumulating evidence suggests that they promote metastasis and the development of metastatic tumor foci [231]. A recent pan-cancer meta-analysis determined that lung cancer patients with higher tumor densities of Foxp3^+^ Tregs had significantly poorer disease-free survival rates [232]. Furthermore, TI-Tregs in patient lung tumors exhibit a distinct transcriptional program marked by elevated Foxp3 expression and increased levels of the Treg-related transcription factors IKZF4, IRF4, SATB1, and GATA1, which maintain chromatin organization and lineage stability [201]. A clinical study of lung tumor observed that Treg levels in peripheral blood also increased with disease stage and were highest in patients with metastatic tumors [233]. Consistently, Kras transgenic mice deficient in Foxp3^+^ Tregs developed significantly fewer lung tumors [234]. Further, murine models of lung adenocarcinoma have demonstrated that Tregs may inhibit CD8^+^ T cell-mediated anti-tumor immunity [231]. The depletion of Tregs led to lung tumor cell death and elevated levels of granzyme A, granzyme B, perforin and IFN-γ in infiltrating CD8^+^ T cells at early stages of tumorigenesis.

Treg-intrinsic or tumor-derived non-coding RNAs are often dysregulated in lung cancer. Treg-intrinsic *miR-155* are Foxp3-programmed regulators that maintain Treg stability and fitness, which is often upregulated in lung cancer where it contributes to tumorigenesis and progression by affecting immune responses and inflammatory processes [235,236]. Antagonizing *miR-155* suppresses tumor progression in lung tumor xenograft models [237]. High *miR-155* levels correlate with poorer overall and disease-free survival, which is partly due to the downregulation of SOCS1, SOCS6, and PTEN [237]. On the tumor-derived side, cancer-cell vesicular miR-214 promotes Treg expansion in vivo, and elevated miR-141 in malignant pleural effusions recruits Tregs via a CXCL1–CXCR2 axis, correlating with poorer outcomes [238,239]. Complementing these, three Treg-related lncRNAs are dysregulated in lung tumors and merit biomarker exploration: (i) *MAFTRR*, a chromatin-associated Th1 lncRNA that promotes Th1 differentiation by suppressing MAF [129,130], intersects with macrophage programming where myeloid-specific MAF deletion reduces tumor size and boosts T-cell antitumor activity [240]; (ii) *TH2-LCR* coordinates Th2 cytokine genes and aligns with observations that Th2 and Tregs dominate peritumoral tertiary lymphoid structures in NSCLC [131]; and (iii) *IFNG-AS1* enhances IFN-γ in T and NK cells with downstream IFN-γ–associated chemokines proposed as metastasis biomarkers [133]. As tumor-infiltrating Tregs are markedly enriched in lung cancer, a stable Treg identity depends on the demethylated Foxp3 TSDR, which preserves Foxp3 expression and suppressive function; this demethylated state is maintained in tumor-infiltrating Tregs from lung tumor patients and is reinforced by tumor-derived TGF-β and hypoxia-driven signaling [241,242]. Histone modifications further shape this Treg-permissive niche: EZH2 is highly expressed in lung cancer and drives the H3K27me3-mediated repression of immune-stimulatory pathways, contributing to diminished CD8^+^ T-cell infiltration and increased Treg dominance [243,244]. Collectively, these results showed that Treg epigenetic regulation reinforces a Treg-skewed microenvironment in lung cancer and highlights testable biomarker and therapeutic targets.

### 4.4. Breast Cancer

Breast cancer is characterized by the uncontrolled proliferation of abnormal mammary epithelial cells, leading to tumor formation and potential metastasis. Notably, the intratumoral accumulation of Tregs is strongly correlated with adverse clinical outcomes, including decreased overall survival [245,246]. This association highlights the pivotal role of Tregs in breast cancer progression, immune suppression within the TME, and response to therapy [247]. Transcriptomic analyses further revealed that Tregs exposed to the TME acquire a distinct “educated” phenotype, which is characterized by the upregulation of chemokine receptors such as CCR8 and CCR4, as well as suppressive molecules including CTLA4 and ICOSs, enabling their recruitment to metastatic niches and the maintenance of local immunosuppression. Tumor-educated Tregs actively drive breast-cancer metastasis rather than serving as passive immunosuppressive bystanders [248]. An integrated scRNA-seq and transcriptomic analysis identified Treg-associated prognostic signatures (*TBC1d4*, *PMAIP*, *IFN-γ*, *LEF1*, *MZB1* and *EZR*) for patients with breast cancer that correlate with survival, immune infiltration, and therapeutic sensitivity [247].

Dysregulated miRNAs and lncRNAs contribute to breast cancer pathogenesis by promoting immunosuppression and supporting tumor growth. *miR-126* overexpression induces the generation of Treg and enhances their function by targeting p85b and affecting the PI3K/Akt pathway [104]. Furthermore, silencing *miR-126* impairs the suppressive function of Tregs in vivo and effectively enhances the anti-tumor effect of CD8^+^T cells in adoptive cell transfer assay using a murine breast cancer model [104]. MiRNAs are also involved in other regulatory functions of Tregs in the TME. Tumor-derived miR-214 can be transported to CD4^+^ T cells, promoting Treg expansion by downregulating the expression of PTEN. These miR-214-induced Tregs facilitate tumor growth and proliferation by producing IL-10 [239]. *MiR-21* is highly expressed in CCR6^+^ Tregs isolated from breast cancer tissues, and in vitro studies show that *miR-21* inhibits the proliferation of CCR6^+^ Tregs by targeting PTEN and then activating the AKT pathway [249]. An increased expression of miR-155 in breast cancer correlates with higher tumor grade, advanced disease stage, and lymph node metastasis [250]. Experimental studies in breast cancer cell lines and xenograft models demonstrate that *miR-155* drives tumor growth by promoting cell proliferation, inhibiting apoptosis, and mediating TGFβ-induced EMT, highlighting its role as a potential therapeutic target. Beyond miRNAs, Treg-associated lncRNAs including *SNHG1*, *SNHG16*, *TH2-LCR* and *MAFTRR* are also highly expressed in breast cancer tissue compared with non-tumoral tissues [115,128,251]. lncRNA *SNHG1* was identified in breast cancer-infiltrating CD4^+^ T cells, and it binds *miR-448*, resulting in decreased IDO expression and increased Foxp3 and IL-10 levels in Tregs. SiRNA-mediated knockdown of SNHG1 reduces tumor progression, indicating that *SNHG1* promotes tumor immune escape by stimulating Tregs differentiation [115,116]. Additionally, the downregulation of *SNHG1* inhibits breast cancer cell proliferation, migration, invasion, and EMT processes while promoting apoptosis in vitro [251]. In vivo *SNHG1* suppression reduces the *miR-641* level, which in turn inhibits breast cancer growth, migration, invasion while inhibiting apoptosis [251,252]. Collectively, these mechanisms contribute to a strongly potent immunosuppressive TME that promotes breast cancer and worsens clinical outcomes.

### 4.5. Glioblastoma

Glioblastoma (GBM) is an aggressive, fast-growing brain tumor that develops from astrocytes, which are the support cells of the central nervous system. Classified as a grade IV astrocytoma, it infiltrates the surrounding brain tissue, making complete surgical removal difficult and leading to frequent recurrence. This disease harbors an immunosuppressive TME with abundant Foxp3^+^ Tregs [253,254]. Circulating Tregs also have been elevated in the blood of GBM patients compared with healthy controls [255], reinforcing the systemic and local immunosuppressive milieu associated with this disease. Treg-associated lncRNA *HOXA-AS2* is significantly upregulated in both glioma biopsy specimens and glioma cell lines [126]. In addition, lncRNA *HOXA-AS2* has been shown to upregulate KDM2A by suppressing *miR-302a*, which in turn increases the expression of Jagged-1, a ligand of notch signaling, thereby promoting GBM progression as well as Treg proliferation and immune tolerance [115]. Furthermore, in vivo studies demonstrated that silencing lncRNA *HOXA-AS2* suppresses Treg proliferation, leading to a reduced secretion of TGF-β and IL-10 and ultimately decreasing immune tolerance in glioma. Additional non-coding RNAs *miR-21*, *miR-146*, *miR-155* and *miR-17-92* clusters, lncRNAs including *HOTAIR*, *SNHG1*, *MALAT1*, and *HIF1A-AS2*, and histone-modifying enzyme EZh2 are also significantly enriched in glioblastoma and linked to GBM prognosis [256,257,258,259,260,261,262,263,264]. However, direct evidence that these regulators increase Treg infiltration or Treg stability in GBM remains limited; current support is stronger at the tumor-cell than at the Treg-specific level.

### 4.6. Pancreatic Ductal Adenocarcinoma

Pancreatic ductal adenocarcinoma (PDAC) is a highly aggressive cancer that is often diagnosed late and is characterized by dense tissue fibrosis and a strongly immunosuppressive tumor environment. These factors contribute to fast tumor growth, early spread, and poor survival. A key feature of PDAC is the presence of Tregs, which the tumor actively attracts and expands to create an immune-protected environment that supports its growth [265,266,267]. Epigenomic profiling in KRAS-driven and KRAS/TP53-mutant mouse models revealed that the tumor-intrinsic activation of Fosl2 enhances Ccl28 expression, creating a chemokine signal that draws Tregs into the tumor; blocking Ccl28 reduces Treg accumulation and suppresses tumor progression [268]. Single-cell RNA-seq analyses of human PDAC further demonstrate that Tregs accumulate early during tumorigenesis and maintain a highly immunosuppressive phenotype characterized by the expression of molecules such as CTLA4 and TIGIT [269]. Another mechanism involves a subset of PDAC cells that aberrantly express cancer-cell Foxp3, which directly activates Ccl5 transcription [270]. This Foxp3-Ccl5 axis promotes Treg recruitment and is positively correlated with Treg abundance in patient tumors, while Ccl5 neutralization in mouse models decreases Treg infiltration and inhibits tumor growth. Together, these studies show that PDAC actively orchestrates Treg recruitment through chemokine pathways, reinforcing an immunosuppressive microenvironment, although much of the current research remains centered on tumor-driven mechanisms rather than in-depth Treg biology. In PDAC, several non-coding RNAs including *miR-17-92*, *miR-21*, *miR-301-3p*, *miR-155*, and LncRNAs *SNHG1*, and *MALAT1* as well as EZh2, HDAC1 and KDM1A are frequently upregulated [271,272,273,274,275,276,277,278,279,280,281]. These alterations lead to global chromatin compaction and the repression of a tumor-suppressive and immune-stimulatory gene program. Although PDAC is characterized by a highly immunosuppressive microenvironment through recruiting Tregs, direct causal evidence for the epigenetic regulation of Tregs in PDAC remains unclear.

## 5. Potential Therapeutic Targets of Tregs in Cancer

Targeting epigenetic regulators of Tregs represents a highly promising strategy to destabilize their suppressive phenotype and improve anti-tumor immunity across various solid tumor including melanoma, breast cancer, lung cancer, PDAC, and GBM. Modern Treg-directed strategies now move beyond earlier systemic depletion, which carries the risk of inducing systemic autoimmunity, and instead focus on the precise modulation of intratumoral Tregs. These approaches include CTLA4-mediated Treg depletion, CCR4/CCR8 blockade to limit Treg recruitment, an induction of Treg fragility, and metabolic disruption to weaken Tregs’ suppressive function [202,282,283,284,285]. The selective depletion of Tregs dramatically reduced lymph-node and lung metastases, despite minimal effects on primary tumor growth, supporting therapeutic strategies that target Treg depletion or functional modulation to improve clinical outcomes [286,287]. Furthermore, *miR-126* has been shown to sustain the suppressive function of Tregs, providing a novel insight into the development of therapeutic strategies for promoting T-cell immunity by regulating Tregs through targeting specific miRNAs. Checkpoint molecules strongly influence Treg activity within tumors, helping to maintain an immunosuppressive microenvironment. Blocking these pathways can weaken Treg suppression and enhance anti-tumor immune responses [288,289,290]. Emerging work also highlights the therapeutic potential of targeting epigenetic modifiers such as EZh2 and HDAC6 to destabilize Tregs’ identity and suppressive function within the TME [77,291,292,293]. Moreover, preclinical studies combining EZH2 inhibitors (e.g., CPI-1205) with checkpoint blockade (anti-CTLA4) show synergistic effects: targeting EZH2 in Tregs enhances the efficacy of CTLA4 therapy without triggering systemic autoimmunity [294]. By selectively weakening intratumoral Tregs through these mechanisms rather than broadly depleting them, we can preserve peripheral tolerance while reinvigorating and enhancing effector T-cell responses [289,295,296]. Such metabolic–epigenetic intervention offers a more precise therapeutic approach to remodel the tumor macroenvironment, overcome immunotherapy resistance, and strengthen the durability of antitumor immunity across multiple solid tumors [289,295,296]. Although each cancer type presents unique microenvironmental barriers, from PDAC’s dense stroma to the immune-privileged constraints of GBM, the overarching concept remains consistent: strategic intertumoral Treg modulation, including metabolic and epigenetic reprogramming, holds significant promise for improving therapeutic outcomes in these hard-to-treat malignancies.

## 6. Conclusions and Perspective

Epigenetic regulation is crucial for the identity, stability, and function of Tregs. DNA methylation, histone modifications, chromatin accessibility, and non-coding RNA networks work together to preserve Foxp3 expression and maintain the transcriptional programs that define Treg lineage commitment, stability and functionality. Tregs are essential for maintaining immune homeostasis and tolerance: their accumulation and activity within tumors can promote immune evasion and tumor progression, which has driven the development of therapeutic strategies aimed at modulating Tregs’ function to enhance anti-tumor immunity. These approaches include antibody-mediated depletion, small molecule inhibitors, ionizing radiation, and exosome-based interventions. However, broad or non-specific Treg targeting carries significant risks, including systemic immune dysregulation and autoimmunity. In this context, cancer type-specific epigenetic reprogramming together with the targeted modulation of Foxp3 stability may offer a safer and more effective strategy by reshaping the intratumoral Treg transcriptional program and function while preserving peripheral tolerance. In this review, we have discussed recent advances in the epigenetic mechanisms that support Tregs stability and function as well as their role in cancer development and progression across multiple tumor types.

Despite substantial progress in elucidating the epigenetic logic that governs Treg biology, important limitations and knowledge gaps remains. These are particularly evident in solid tumors, where Tregs acquire unique, tumor-adapted epigenetic and metabolic programs that are not fully captured by studies of circulating or lymphoid tissue-derived Tregs. Therapeutic translation is further complicated by challenges, such as achieving a selective intratumoral Tregs modulation without compromising systemic immune balance, overcoming epigenetic plasticity and pathway redundancy, ensuring durable therapeutic responses, and optimizing the dosing, delivery, and specificity of epigenetic agents. Moreover, tumor heterogeneity and dynamic interactions within the TME can limit the predictability and durability of Treg-targeted interventions. Nevertheless, rapid advances in emerging technologies are beginning to address these challenges. Single-cell and spatial multi-omics approaches are providing an unprecedented resolution of Treg heterogeneity, lineage dynamics, and tumor-specific epigenetic states, while innovations in spatial epigenomics, epigenetic drug design, biomarker-guided patient stratification, and targeted delivery platforms are improving therapeutic precision. Together, these developments are paving the way for the rational design of next-generation Treg-directed epigenetic therapies. The continued integration of mechanistic insights with translational and clinical studies will be essential to fully harness the therapeutic potential of the epigenetic modulation of Tregs in cancer while minimizing adverse effects on immune tolerance.

## Data Availability

No new data were created or analyzed in this study. Data sharing is not applicable to this article.
